# Evaluating two implant designs in patients undergoing primary total knee arthroplasty using a novel measure of early optimal recovery: a retrospective observational study

**DOI:** 10.1007/s12306-024-00851-z

**Published:** 2024-08-02

**Authors:** L. Z. van Keulen, R. J. A. Sonnega, N. R. A. Baas, T. Hogervorst, C. Muehlendyck, P. Bourras, T. A. J. ten Kate, T. Galvain, S. Dieleman, P. M. van Kampen

**Affiliations:** 1 Department of Orthopedics Rijswijk, Bergman Clinics, Braillelaan 10, 2289 CM Rijswijk, The Netherlands; 2 Johnson & Johnson Medical, 1 Rue Camille Desmoulins, 92130 Issy Les Moulineaux, France; 3 Johnson & Johnson Medical BV, Computerweg 14, 3821 AB Amersfoort, The Netherlands; 4 Department of Science and Innovation, Bergman Clinics, Gooimeer 11, 1411 DE Naarden, The Netherlands; 5 Johnson & Johnson Medical, Johnson & Johnson MEDICAL GmbH, Hummelsbütteler Steindamm 71, 22851 Norderstedt, Germany; 6 Department Business Intelligence, Bergman Clinics, Gooimeer 11, 1411 DE Naarden, The Netherlands

**Keywords:** Prosthesis implantation design, Total knee arthroplasty, Outcomes research, Value of healthcare

## Abstract

**Purpose:**

Quality of care in total knee arthroplasty (TKA) between implants was assessed using a novel composite outcome measure, early optimal recovery (EOR), to indicate ideal clinical outcomes and minimal healthcare resource utilization.

**Methods:**

Patients that underwent primary TKA in the study group (ATTUNE® Knee System) or control group (LCS® COMPLETE Knee System) were included in this retrospective, single-center study. EOR was defined as no complications, no readmissions, no extra outpatient visits, ≤ 48 h length of hospital stay (LOS), and restored range of motion and pain perception at 3-month follow-up. Multivariate logistic regression was used to compare EOR between the study and control groups. Results were adjusted for differences in baseline characteristics and are presented with 95% confidence intervals (CI). Data were collected from a specialized clinic for elective surgeries in the Netherlands, between January 2017 and December 2020.

**Results:**

A total of 566 patients (62.4% female, mean age 67 years) were included for analysis; 185 patients (32.7%) underwent TKA in the study group. Compared to the control group, patients in the study group had greater probability of achieving EOR (65.8% [95% CI: 55.1–75.2] vs. 38.9% [95% CI: 32.8–45.3]; *p* < 0.001), a LOS ≤ 48 h (77.2% [95% CI: 67.7–84.5] vs. 61.4% [95% CI: 54.7–67.7]; *p* < 0.05), and ideal pain perception at 3-month follow-up (93.3% [95% CI: 85.7–97.0] vs. 78.2% [95% CI: 71.0–83.9]; *p* < 0.05).

**Conclusion:**

The study group was associated with a greater probability of achieving EOR versus the control group, suggesting improved quality of care.

**Supplementary Information:**

The online version contains supplementary material available at 10.1007/s12306-024-00851-z.

## Introduction

### Background

Total knee arthroplasty (TKA) is a widespread surgery, capable of recovering articular function and relieving pain in patients with osteoarthritis [1]. Incidence of TKA has been rising steadily over time, from just over 7000 surgeries performed in the Netherlands in 2007 to more than 25,000 performed in 2019 [2]. As more than 97% of TKA are performed on patients aged > 50 years, increasing life expectancy coupled with population growth in the Netherlands is expected to further increase the incidence of TKA over time [2, 3].

Key performance metrics in TKA frequently include number of postoperative complications, functional outcomes such as range of motion (ROM), and patient-reported pain and satisfaction using scores such as the Oxford knee score (OKS) and knee society score (KSS) [1, 4]. While TKA is associated with positive outcomes, approximately 20% of patients report dissatisfaction regarding pain and functionality following the procedure [4–6]. Additionally, health resource utilization (HRU) in TKA is high, particularly with respect to length of stay (LOS) and operating time [7]. Thus, as the incidence of TKA rises, so does the clinical and HRU burden associated with suboptimal procedures, and the need for quality improvements grows.

Value-based healthcare is a patient-centric model in which the quality of healthcare is measured according to the health outcomes achieved per unit cost [8]. In this model, metrics which provide a comprehensive, accurate reflection of patient satisfaction and quality of life are crucial; however, the complexity of healthcare makes accurate assessment difficult. Evaluation of TKA procedures using specific, individual metrics may fail to capture the extent of the procedure and can lead to significant variability in outcome reporting among studies [9]. All-or-none metrics are binary composite outcomes where overall success requires that a predefined ideal condition is met in each contributing element. By capturing the full complexity of procedures and ensuring that a successful procedure reflects satisfactory outcomes across all aspects of care, these metrics aim to raise the standard of patient-centricity in healthcare [10]. All-or-none metrics have found success in other surgical areas such as gastrointestinal and cardiovascular surgery, but have been studied less in orthopedic surgery [11–13]. In this study, the impact of implant design on quality of care in TKA was investigated using a novel, composite, all-or-none metric, early optimal recovery (EOR). The primary objective of TKA is to reduce pain and improve knee functionality [1, 14, 15]. Thus, EOR was designed to capture patient-reported pain, functionality measures, short-term complications, readmissions, prolonged LOS (> 48 h), and additional outpatient visits.

In this study, the ATTUNE® Knee System (study group) was compared against the LCS® COMPLETE Knee System (control group). The implant of the study group was developed to improve joint kinematics, reduce pain, increase stability, and address implant fit, and has previously demonstrated favorable postoperative clinical outcomes [4, 16–23], as well as decreased HRU burden in the form of reductions in LOS, number of outpatient visits, and complication rate compared to patients treated with other implants [17, 24–28].

### Objectives

The objective of this study is to evaluate the overall quality of care achieved in the study group compared to the control group using a novel, composite, all-or-none metric called EOR. The study aims to determine if there is a significant difference in EOR between the two TKA implants, hypothesizing that the study group will show superior results over the control group.

## Materials and methods

### Study design

This retrospective study used EOR to evaluate two knee implants, the ATTUNE® Knee System (study group) and the LCS® COMPLETE Knee System (control group), at the Orthopedic Center, Bergman Clinics, Rijswijk, The Netherlands. Both implant designs were manufactured by DePuy Synthes, Warsaw, IN. The institutional review board of Bergman Clinics approved this study (BMC2020-008) and the Medical Ethics Committee of Nieuwegein deemed the study not subject to the Dutch Medical Research Involving Human Subjects Act (WMO) (W21.194). Data were extracted from patients’ medical records and anonymized by the physician (surgery date, patient name, and patient ID were deleted and variables were dichotomized when possible). Due to the anonymization strategy and as per general data protection regulation (GDPR), patients’ informed consent was not required.

All procedures were performed by three experienced orthopedic surgeons, each of whom performed more than 60 previous TKA procedures. The surgeries were performed in the Bergman Clinics, a large private clinic organization with more than 70 clinics in the Netherlands. TKA procedures were done in 5 separate clinic locations. One surgeon’s experience was primarily with the study device, one the control device, and one frequently implanted both devices. The study device was introduced in February 2019; prior to this, all surgeons utilized the control device.

### Participants

Patients aged ≥ 18 years, with a body mass index (BMI) < 35 kg/m^2^ and an American Society of Anesthesiologists (ASA) score < III that underwent a unilateral primary TKA between January 2017 and December 2020 were included in this study. Exclusion criteria were contralateral TKA, infection or fracture of the ipsilateral knee < 1 year before TKA surgery, previous surgery within the same surgical area < 90 days before TKA surgery or contralateral TKA < 3 months after the index TKA. Uncemented TKA procedures, and procedures in which osteosynthesis material was removed, or in which a patella button or tibial sleeve component was used, were excluded. The implant used was determined by availability and surgeon preference and not influenced by patient characteristics.

### Knee implant systems

For a schematic illustration of both prosthetic systems, please refer to Fig. 1 of Koster et al. [29]. Both knee implant systems utilize biocompatible materials such as cobalt-chromium alloys and ultrahigh molecular weight polyethylene for durability and reduced wear. Both aim to restore knee function, alleviate pain, and improve the overall quality of life for patients [29]. They focus on providing stability, range of motion, and longevity of the implant. In the current study, all surgeons used devices with a rotating platform, which were cemented, and cruciate-stabilized.

**Fig. 1:**
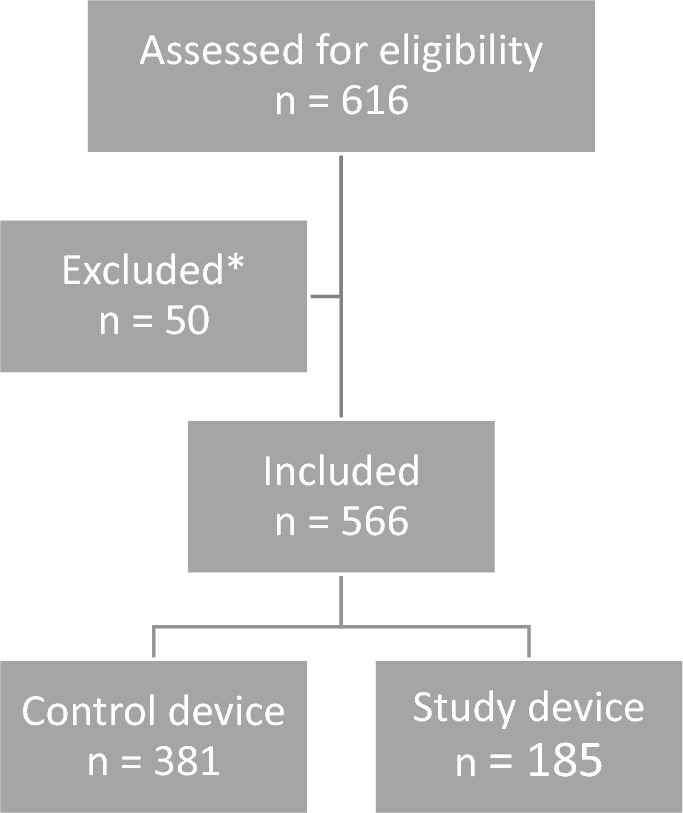
Inclusion flowchart. *Exclusion due to the use of a patella button (*n* = 1) or a tibial sleeve component (*n* = 12), due to removal of osteosynthesis material during the TKA surgery (*n* = 2) or due to contralateral TKA surgery one year prior (n = 34) or less than three months after (*n* = 1) the TKA surgery. TKA: total knee arthroplasty

### Control

The LCS® COMPLETE Knee system is known for its mobile-bearing design, allowing rotation and translation that mimic natural knee movements [30]. This system focuses on providing high conformity and reducing wear by allowing for self-alignment [30].

### Study

The ATTUNE® Knee system emphasizes kinematic functionality and patient-specific biomechanics. It is designed to enhance knee stability in mid-flexion by featuring a continuously changing radius of femoral component curvature. This articulation surface geometry aims to encourage gradual femoral rollback, thereby reducing surface stresses [4, 16–23]. The bone-implant interface of the femoral component has fewer ridges compared to the control devices, providing a larger contact area. The study device also has more and smaller sizing increments available [29].

### Surgical procedure

After administering either spinal or general anesthesia and prophylactic antibiotics, a tourniquet was placed around the upper leg of the patient. The standard medial parapatellar approach was used to access the joint. After measuring the extramedullary tibial alignment and performing a proximal tibial osteotomy, an intramedullary rod was placed to determine the femoral alignment and femoral osteotomy was performed. In case of asymmetry, osteophytes were removed and/or the varus-valgus angle was adjusted and/or medial/lateral collateral ligament release was performed. When flexion and extension gaps corresponded, final osteotomies were performed, and the knee implant component was cemented.

All patients received identical healthcare, and except from a change in anesthesia method, they had identical recovery programs throughout the period of observation. The anesthesia method was changed in September 2019 from a femoral block with a low dose of local infiltration anesthesia (LIA) to a saphenous block and a high dose of LIA.

All patients were admitted on the day of surgery. Patients were mobilized by a physiotherapist when the effect of anesthesia had worn off. Patients were discharged when (1) they were able to walk independently with crutches, (2) their pain was under control with oral medications, and (3) they had no wound complications. The standard pain protocol included paracetamol, nonsteroidal anti-inflammatory drugs, tramadol, and gabapentin.

### Outcomes

Patient demographical characteristics included age, sex, BMI, smoking status, and ASA classification. Clinical characteristics included comorbidities (rheumatic diseases, cardiac disorder or coronary heart disease, hypertension, chronic obstructive pulmonary disorder, history of cerebrovascular accident, and diabetes mellitus) as well as previous surgeries in the operated knee.

The primary outcome measurement was the proportion of patients achieving EOR following TKA. EOR was assessed at 3-month follow-up and defined as no negative outcome measures (< 48 h LOS following initial surgery, no outpatient visits other than the 2 regular follow-up visits, no surgery-related readmissions, and no surgery-related complications), ideal ROM (complete extension [0 degrees] and complete flexion [≥ 120°] or flexion/extension greater or similar to the preoperative flexion/extension [31]), and ideal pain perception (a negative answer to the question: “do you still feel pain in the operated knee?” [14]). Complications included bleeding, wound nonhealing (such as dehiscence or necrosis), wound infection, deep joint infection, thromboembolic event, neurovascular injury, joint instability, ligament injury, malalignment, fracture, tibiofemoral or patellofemoral dislocation, implant loosening, implant fracture/tibial insert dissociation, bearing surface wear, osteolysis, reoperation, revision, or death. Baseline characteristics were adjusted for covariates (BMI, ASA classification, previous surgery, surgeon, and anesthesia methods) to account for differences between groups.

Secondary, individual outcome measurements were compared between the two groups including: ROM, pain perception, complication rate and type, total number of outpatient visits, ≤ 48 h LOS.

### Sample size

We anticipated an EOR percentage of 75–80% in the study group and 60% in the control group. With an alpha of 5%, a power of 90%, and a two-sided *Z*-test, with ratio 1:2, the sample size calculation indicated approximately 170 in the study group and 433 in the control group.

### Statistical methods

Outcome measures were analyzed descriptively. Values are reported with standard deviations (SDs) or 95% confidence intervals (CI). Bivariate comparison of baseline data was conducted.

Multivariate models were used to examine the outcomes between the study group and control group and were adjusted for the relevant demographic, patient, and procedural characteristics, including anesthesia method. Logistic regression models were used for categorical outcomes. Generalized linear modeling was used for length of stay with gamma distribution and log link. Confounders were included either on the basis of an absolute standardized difference of > 0.10 between the two groups, or by expectations from the literature. There was a check for multicollinearity.

All analyses were conducted using R Studio (2021.09.0 Build 351). Statistical significance was set at *p* < 0.05.

## Results

### Participants

A total of 566 patients were included in this study, of which 185 (32.7%) patients were included in the study group. See Fig. 1 for the inclusion flowchart.

The overall mean (SD) age of the cohort was 67 (8) years and 62.4% of the patients were female. All other baseline patient characteristics are found in Table 1. Most baseline characteristics were similar between the study and control groups. However, significant differences were observed in ASA II status (72.4% vs. 25.7%; *p* < 0.001), Charnley score A (38.4% vs. 20.5%; *p* < 0.001), and previous knee surgeries (32.4% vs. 8.1%; *p* < 0.001) (Table 1).

**Table 1:** Baseline patient characteristics

	Overall (*n* = 566)	Control group (*n* = 381)	Study group (*n* = 185)	*p* value
Female, n (%)	353 (62.04%)	242 (63.5%)	111 (60.0%)	0.473
Age, years (SD)	67 (8)	67 (8)	67 (8)	0.632
BMI, m/kg^2^ (SD)	28.1 (3.7)	27.9 (3.7)	28.5 (3.5)	0.071
ASA				
ASA I, n (%)	334 (59.0%)	283 (74.3%)	51 (27.6%)	< 0.001
ASA II, n (%)	232 (41.0%)	98 (25.7%)	134 (72.4%)	–
Current smoker, n (%)	51 (9.0%)	33 (8.7%)	18 (9.7%)	0.795
Diagnosis				
Osteoarthritis, n (%)	563 (99.5%)	381 (100%)	182 (98.4%)	0.035
Posttraumatic, n (%)	2 (0.4%)	0 (0%)	2 (1.1%)	–
Comorbidities, n (%)	226 (40.0%)	154 (40.4%)	72 (38.9%)	0.802
Previous surgery, n (%)	91 (16.1%)	31 (8.1%)	60 (32.4%)	< 0.001
Charnley Score				
A, n (%)	149 (26.3%)	78 (20.5%)	71 (38.4%)	< 0.001
B1, n (%)	326 (58.6%)	236 (61.9%)	90 (48.6%)	–
B2, n (%)	82 (14.5%)	64 (16.8%)	18 (9.7%)	–
C, n (%)	9 (1.6%)	3 (0.8%)	6 (3.2%)	–

*ASA* American Society of Anesthesiologists, *SD* standard deviation

### Procedural characteristics

The study device was only used in TKA performed in 2019 and 2020, while the number of TKA with the control device decreased over time. Surgeon 1 performed relatively fewer operations in the study group than the control group (32.4% vs. 83.7%; *p* < 0.001) (Table 2). Due to a change in anesthesia method, the femoral block + LIA was used less often in the study group than in the control group (48.6% vs. 96.9%; *p* < 0.001).

**Table 2:** Procedural characteristics

	Control group (*n* = 381)	Study group (*n* = 185)	*p* value
Side left, n (%)	167 (43.8%)	94 (50.8%)	0.141
Surgery year			
2017, n (%)	42 (11.0%)	0 (0.0%)	< 0.001
2018, n (%)	211 (55.4%)	0 (0.0%)	–
2019, n (%)	123 (32.3%)	110 (59.5%)	–
2020, n (%)	5 (1.3%)	75 (40.5%)	–
Surgeon			
Surgeon 1, n (%)	319 (83.7%)	60 (32.4%)	< 0.001
Surgeon 2, n (%)	60 (15.7%)	10 (5.4%)	–
Surgeon 3, n (%)	2 (0.5%)	115 (62.2%)	–
Anesthesia method			
Fem block + LIA, n (%)	369 (96.9%)	90 (48.6%)	< 0.001
LIA, n (%)	11 (2.9%)	34 (18.4%)	–
Sap block + LIA, n (%)	1 (0.3%)	61 (33.0%)	–

*Fem* femoral, *LIA* local infiltration anesthesia, *Sap* saphenous, *SD* standard deviation

Surgeon 3 only performed TKA in 2019 and 2020, and performed a greater proportion of surgeries on ASA II patients compared to surgeons 1 and 2. Surgeon 3 also operated on more patients with previous ipsilateral knee surgery (*p* < 0.001), and had more participants in the study group (*p* < 0.001) than surgeons 1 and 2.

There were moderate to high correlations for the following variables: implant design, surgery year, anesthesia method, surgeon, ASA classification, and previous surgery. Due to the differences in baseline values, results were adjusted for covariates (BMI, ASA classification, previous surgery, surgeon, and anesthesia methods) unless specified otherwise. Operating year could not be integrated due to multicollinearity.

### Primary outcome: EOR

**Fig. 2:**
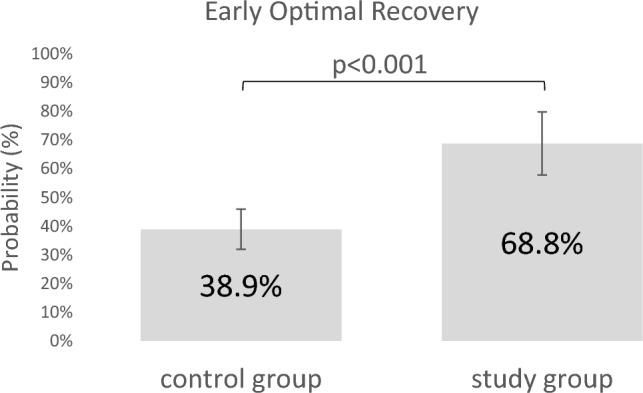
Probability of achieving EOR. Probability of achieving EOR in the control group (*n* = 381) versus the study group (*n* = 185). Probabilities were adjusted for covariates (BMI, ASA, previous surgery, surgeon, and anesthesia methods). ASA: American Society of Anesthesiologists, BMI: body mass index, EOR: early optimal recovery

After adjusting for covariates, patients in the study group had a significantly greater probability of achieving EOR (65.8% [95% CI: 55.1–75.2]) compared to patients in the control group (38.9% [95% CI: 32.8–45.3]; odds ratio: 0.33; *p* < 0.001, Table 3, Fig. 2). Unadjusted results of EOR are presented in Supplementary Table S1.

**Table 3:** Adjusted probability of study outcomes

Outcome, % (95% CI)	Control device (*n* = 381)	Study device (*n* = 185)	OR	*p* value
LOS ≤ 48 h	61.4 (54.7–67.7)	77.2 (67.7–84.5)	0.47	0.018
Ideal ROM	99.4 (96.2–99.9)	98.8 (91.9–99.8)	1.86	0.639
Pain-free	78.2 (71.0–83.9)	93.3 (85.7–97.0)	0.26	0.016
≤ 2 outpatient visits	84.3 (78.8–88.5)	89.9 (81.7–94.7)	0.60	0.273
Readmissions	2.1 (0.9–4.6)	2.2 (0.6–7.2)	0.96	0.962
Complications	3.6 (1.9–6.9)	2.3 (0.7–7.4)	1.59	0.555
**EOR**	**38.9 (32.8–45.3)**	**65.8 (55.1–75.2)**	**0.33**	**< 0.001**

*CI* confidence interval, *EOR* early optimal recovery, *LOS* length of stay, *OR* odds ratio, *ROM* range of motion

### Secondary outcomes

Each individual outcome contributing to EOR was adjusted for covariates and compared between groups (Table 3). A significantly larger proportion of patients had a < 48 h LOS in the study group and were pain-free at 3 months in the study group compared to the control group. Differences in complication rates, readmission rates, number of outpatient visits, number of patients with ≤ 2 outpatient visits, and number of patients achieving ideal ROM were not significant between groups (Table 3). Complications observed in this study are presented in Table 4, and unadjusted study outcomes are presented in Supplementary Table S1.

**Table 4:** Type of complication

Complication type, n (%)	Control device (*n* = 381)	Study device (*n* = 185)
Thromboembolic event	4 (1.0%)	1 (0.5%)
Bleeding	2 (0.5%)	1 (0.5%)
Fracture^a, b^	1 (0.2%)	1 (0.5%)
Partial rupture patellar tendon	1 (0.2%)	–
Arthrofibrosis^b^	2 (0.5%)	–
Infection^b^	1 (0.2%)	–
Thrombophlebitis	-	1 (0.5%)
Insert revision^b, c^	1 (0.2%)	–
Bladder retention	1 (0.2%)	1 (0.5%)
Mobilization of knee on OK	2 (0.5%)	1 (0.5%)
Gastrointestinal bleeding^d^	1 (0.2%)	
Transient ischemic attack		1 (0.5%)
**Total**	**16 (4.2%)**	**7 (3.2%)**

^a^Included a traumatic patellar fracture and a perioperative femur condyle fracture

^b^Followed by re-surgery

^c^Due to instability

^d^Due to use diclofenac

### Other analyses

Following the primary analysis, several subgroup analyses were also conducted: (1) only including years 2019–2020, as these years included both implants while in 2017–2018 only the control device was used, and because a decreasing trend in LOS was observed throughout, (2) excluding saphenous block + LIA as this was mostly used in 2020, mainly by surgeon 3 and mostly alongside the study device, (3) excluding surgeon 3 who operated mostly with the study device, (4) only including year 2019 with the same justification as (1) and because in 2019 there was an equal distribution of procedures between the control and study devices, and (5) excluding surgeons 2 and 3, and saphenous block + LIA with the same justification as (2) and (3), and because surgeon 1 did not use saphenous block + LIA except in one case. In each of the subgroup analyses, patients in the study group were significantly more likely to achieve EOR than those in the control group (all *p* < 0.05, Table 5).

**Table 5:** Subgroup analyses of EOR

Subgroup analysis, % (95% CI)	Control device (*n* = 381)	Study device (*n* = 185)	OR	*p* value
Only including years 2019–2020	34.3 (24.7 − 45.5)	60.5 (51.4–68.9)	0.34	0.002
Excluding sap block + LIA	42.8 (37.0–48.7)	68.6 (56.7–78.4)	0.34	< 0.001
Excluding surgeon 3	44.1 (38.8–49.6)	69.8 (56.4–80.5)	0.34	< 0.001
Only including year 2019	38.7 (29.1–49.3)	66.7 (55.3–76.4)	0.32	0.002
Excluding surgeons 2–3 and sap block + LIA	46.0 (40.3–51.9)	74.7 (61.2–84.7)	0.29	< 0.001
All patients	38.9 (32.8–45.3)	65.8 (55.1–75.2)	0.33	< 0.001

*CI* confidence interval, *EOR* early optimal recovery, *LIA* local infiltration anesthesia, *OR* odds ratio, *Sap* saphenous

## Discussion

All results were adjusted for covariates including BMI, ASA classification, previous surgery, surgeon, and anesthesia method. The percentage of patients who achieved EOR was significantly higher in the study group (65.8% [95% CI: 55.1–75.2]) than in the control group (38.9% [95% CI: 32.8–45.3]; odds ratio: 0.33; *p* < 0.001). Compared to the control group, patients in the study group also had significant improvements in pain perception (pain-free rate control 78.2% vs. study 93.3%) at 3-month follow-up, and a significantly higher number of patients were discharged from hospital within 48 h after the procedure (LOS ≤ 48 h control 61.4% vs. study 77.2%). Differences between groups in postoperative ROM, complication rates, number of reoperations, and number of patients requiring readmission were not significant.

As a retrospective study, the lack of randomization and the opportunity for bias in measuring outcomes after exposure are inherent limitations of this study. The study reflects the experience of a single-center, and thus the replicability of these results is uncertain. Nevertheless, while we identified difference in baseline characteristics, they were controlled for using multivariate analysis methods. Robustness of the results was also investigated further in subgroup analyses. Additionally, the noticeably greater proportion of control devices compared to study devices, and the small sample size of patients with complications, readmissions, and reoperations place limitations on the conclusions which can be drawn. Due to the retrospective design, it was not possible to include robust measures of patient satisfaction or ambulatory status; future prospective studies may seek to address this.

Composite metrics in orthopedic surgery have found success in two studies where the quality of care was compared between hospitals; the composite metrics were superior to individual outcomes in highlighting variation between centers, and differences were detected faster and required less data, potentially decreasing the time required to introduce procedural innovations [32, 33]. To our knowledge, EOR is the first all-or-none composite outcome that attempts to demonstrate the quality of care associated with two TKA implant devices.

EOR was developed to evaluate the quality of care in TKA by combining functional outcomes (ideal ROM) and indicators of HRU burden (< 48 h LOS, no readmissions, and no reoperations) with measures of patient satisfaction (ideal pain perception). Therefore, EOR provides a simple, comprehensive image of the value of healthcare interventions in TKA and can be used to drive improvement initiatives such as the use of new devices, surgical techniques, or care programs. Additionally, metrics such as EOR could be used to compare performance between surgeons, facilities, or countries, as well as helping patients to make informed decisions about their healthcare.

The hypothesis that there would be a higher EOR in the study group compared to the control group was confirmed in this study. This aligns with previous findings which compared PROMs between the two devices where the study device (although underpowered) showed better three-month postoperative scores in KOOS-PS, OKS, and NRS activity compared to the control device [29]. The other clinical outcomes of Koster et al. [29] however were similar for the two designs and comparable to those reported in the Dutch registry for all patients undergoing TKA [2]. Although the EOR is not completely comparable to the outcomes measured in the study of Koster et al. [29], it does show a higher percentage of patients who are pain-free at 3 months post-surgery in the study group (98.8%) compared to the control group (78.2%; *P* < 0.02). In addition, it shows the advantages on HRU (higher percentages of LOS rate within 48 h for the study group (77.2%) compared to the control group (61.4%; *P* < 0.02). This difference in LOS between the study and control device was also seen in a study of Meermans et al. (2020) where patients who received the study device had a significantly shorter LOS (adjusted mean 2.76 days) versus the control group (adjusted mean 3.43 days; *P* < 0.01) [24]. In this case, it seemed like the design to enhance knee stability in mid-flexion by featuring a continuously changing radius of femoral component curvature helped with increasing the combined outcome EOR. These results align with previous research, where innovations in joint kinematics, implant fit, and stability associated with the study device have demonstrated improvements in pain perception, [4, 18, 34] and led to reductions in hospital LOS compared to patients treated with other implants, [24–28] addressing ongoing challenges in TKA.

The definition of optimal recovery is complex, and subjectivity in measures of patient satisfaction may reduce the alignment of EOR to real-world patient experience [35–38]. Future work may seek to enhance the value of EOR through the inclusion of patient-reported outcome measures such as the visual analog scale and KSS, which could increase the objectivity, applicability, and prognostic accuracy of EOR as an indicator of procedural success [39, 40]. Consensus studies could be used to reach agreement on patient treatment goals, [41] and align EOR with expert opinion on ideal quality of care [42]. Outcomes may be weighted to reflect their impact on patient satisfaction, for example, placing greater emphasis on a reoperation over a LOS > 48 h, and metrics more reflective of real-world outcomes, for example, indicators of joint functionality which better reflect postoperative ambulatory status and patient satisfaction [43].

EOR successfully measured the quality of care associated with TKA procedures and may have value in a value-based healthcare setting. EOR could be used to drive improvement initiatives, standardize measurement, and compare cross-center performance; however, further prospective studies are needed. Participants in the study group had a greater probability of achieving EOR in comparison to the control group, indicating that the implant leads to improved quality of care.

## Supplementary Information

Below is the link to the electronic supplementary material.

Supplementary file 1 (DOCX 69 kb)

## Data Availability

The data that support the findings of this study are available from the corresponding author upon reasonable request. More details on Johnson & Johnson’s commitment to transparency are available via the following link: https://www.jnj.com/covid-19/our-commitment-to-transparency.
