# Supplementary material for: Evaluating two implant designs in patients undergoing primary total knee arthroplasty using a novel measure of early optimal recovery: a retrospective observational study
Source: Musculoskelet Surg. 2024 Aug 2;109(1):71–9. doi: 10.1007/s12306-024-00851-z (PMC11876230; doi:10.1007/s12306-024-00851-z)
Supplement: Supplementary file 1 — (DOCX 69 kb) [file 12306_2024_851_MOESM1_ESM.docx]

# SUPPLEMENTARY FILES

**Supplementary Table 1:** Unadjusted study outcomes

| Outcome, n (%) | Control device  (n=381) | Study device  (n=185) | p value |
| --- | --- | --- | --- |
| LOS ≤48 hours | 213 (55.9%) | 149 (80.5%) | <0.001 |
| Ideal ROM | 341 (89.5%) | 154 (83.2%) | <0.001 |
| Pain-free | 284 (74.5%) | 151 (81.6%) | 0.130 |
| ≤2 outpatient visits | 331 (86.9%) | 148 (80.0%) | 0.045 |
| Readmissions | 11 (2.9%) | 4 (2.2%) | 0.783 |
| Complications | 16 (4.2%) | 7 (3.2%) | 0.994 |
| Reoperations | 6 (1.6%) | 0 (0.0%) | 0.185 |
| EOR | 151 (39.6%) | 96 (51.9%) | 0.006 |

LOS: length of stay, ROM: range of motion, EOR: early optimal recovery.
